# Upregulation of Extracellular Vesicles-Encapsulated miR-132 Released From Mesenchymal Stem Cells Attenuates Ischemic Neuronal Injury by Inhibiting Smad2/c-jun Pathway *via* Acvr2b Suppression

**DOI:** 10.3389/fcell.2020.568304

**Published:** 2021-03-08

**Authors:** Bin Feng, Lei Meng, Liming Luan, Zhihao Fang, Peng Zhao, Guangyu Zhao

**Affiliations:** Department of Neurosurgery, Shandong Provincial Hospital Affiliated to Shandong First Medical University, Jinan, China

**Keywords:** middle cerebral artery occlusion, extracellular vesicles, microRNA-132, activin receptor type-2B, ischemic neuronal injury

## Abstract

Ischemic cerebrovascular disease is a significant and common public health issue worldwide. The emerging roles of mesenchymal stem cells (MSCs)-derived extracellular vesicles (EVs) in ischemic neuronal injury continue to be investigated. The current study aimed to investigate the role of EV-derived miR-132 from MSCs in ischemic neuronal injury. EVs were initially isolated from bone MSCs (BMSCs) and subsequently evaluated. A middle cerebral artery occlusion (MCAO) mouse model was constructed with the neurological function evaluated through a series of neurological scores, a pole test, and a foot fault test. Histopathological changes, neuron viability, and apoptosis, as well as cerebral infarction, were detected by hematoxylin and eosin (HE) staining and 2,3,5-triphenyltetrazolium hydrochloride (TTC) staining. The targeting relationship between microRNA (miR)-132 and Activin receptor type IIB (Acvr2b) was further confirmed based on dual-luciferase reporter gene assay results. Loss- and gain-of-function assays were conducted to elucidate the role of miR-132, EV-derived miR-132, Acvr2b, and Smad2 in oxygen-glucose deprivation (OGD)-treated neurons, and in mice models. Neuronal cell viability and apoptosis were evaluated via Cell Counting kit-8 (CCK-8) and flow cytometry. Our results indicated that Acvr2b was highly expressed, while miR-132 was poorly expressed in the MCAO mice and OGD-treated neurons. Acvr2b silencing or upregulation of miR-132 led to an elevation in neuronal activity, decreased neuronal apoptosis, reduced expression of Bax, and cleaved-caspase 3, as well as increased Bcl-2 expression. Acvr2b expression was targeted and inhibited by miR-132. EV-derived Acvr2b promoted activation of phosphorylated-Smad2 (p-Smad2)/c-jun signaling pathway, ultimately inducing neuronal injury. Our study provides evidence demonstrating that the overexpression of c-jun inhibits the protective role of MSCs-derived EV-miR-132 in neuronal injury. Upregulation of EV-derived miR-132 released from MSCs attenuates ischemic neuronal injury by inhibiting Smad2/c-jun pathways *via* the suppression of Acvr2b.

## Introduction

Tissue injury caused by I/R has been well documented to be a significant cause of morbidity and mortality sprouting from various pathological etiologies, such as myocardial infarction and ischemic stroke (Yu et al., 2019; Mai et al., 2020). Brain injury following cerebral ischemia has been reported as a consequence of various pathological processes, including excitotoxicity, inflammation, and apoptosis (Du et al., 2010; Launey et al., 2020). Cerebral I/R has been demonstrated to induce neuronal death through several biologically feasible pathways (Luo et al., 2020). Research into ischemic neuronal injury therapy continues to be a topic of significant interest. For example, treatment with ischemic premelatonin has been shown to alleviate acute neuronal injury after ischemic stroke (Feng et al., 2017). Combined gene therapy has also been implicated as a mitigating factor in neuronal injury in mice with focal ischemic injury (Molcho et al., 2018).

Various miRs have been implicated in many human disease processes, including ischemic stroke, many of which have been identified clinical diagnostic and therapeutic targets (Zhang et al., 2016). Accumulating evidence has suggested miRs are particularly abundant in brain and play significant roles in the development and maintenance of neuronal phenotypes (Saugstad, 2010). The ability of miR-132 to manipulate major target genes involved in neuronal apoptosis and adaptation induced by neuronal stress has been recently reported (Sessa et al., 2019). The downregulation of miR-132 has been identified in studies involving MCAO mice and OGD-treated neurons, while the overexpression of miR-132 has been suggested to alleviate brain injury in MCAO mice and protect hippocampal neurons from OGD-induced apoptosis (Zuo et al., 2019). Existing literature has documented that bone MSCs (BMSCs)-derived EVs may play a contributory role in the improvement of ischemic neuronal injury while EVs have also been noted to display an abundant expression of miR-132 (Ma et al., 2018; Xiao et al., 2018). MSCs play a crucial regulatory role in cellular homeostasis by secreting exosomes, EVs, and cytokines (Kou et al., 2018). During the current study, miR-132 was predicted to bind to Acvr2b mRNA. Activins are members of TGF-β promoter ligand family which possesses a wide array of biological functions in embryonic stem cells and in differentiated tissues (Olsen et al., 2015). Acvr2b has been previously predicted as one of the downstream targets of miR-132 (Jiang et al., 2015). Overexpression of Acvr2b has been previously detected in neuron injury and has been speculated to aggravate neuron injury (Looney et al., 2017). Furthermore, studies have indicated that Acvr2b promotes the expression of p-Smad2 (Gao et al., 2018). As a notable element in intracellular signal transduction of TGF-β (Gotoh et al., 2020), Smad2 and p-Smad2 have been reported to upregulate the expression of c-jun (Nyati et al., 2011). Transcription factor c-jun responds to JNK and Ras/MAPK signals to control key cellular behavior, including proliferation and apoptosis (Chakraborty et al., 2015). Another study concluded that p-Smad2 and c-jun expression are both upregulated in cerebral I/R injury, which induces neuronal injury (Wang S. et al., 2016). Following the aforementioned exploration of literature, the current study aimed to investigate whether EV-derived miR-132 released from MSCs could attenuate ischemic neuronal injury through regulation of Acvr2/Smad2/c-jun axis.

## Materials and Methods

### Ethical Approval

The study protocol was approved by the Ethics Committee of the Shandong Provincial Hospital and performed in strict adherence with the *Declaration of Helsinki*. All animal experiments were conducted in accordance with *Guide for the Care and Use of Laboratory Animals* published by the National Institutes of Health, and approved by the Animal Ethics Committee of Shandong Provincial Hospital.

### Mouse Model of MCAO

A total of 96 healthy adult male C57BL/6J mice (weighting 22–25 g) were purchased from Shanghai Lab. Animal Research Center. The mice were initally housed in a controlled environment (at a standard temperature of 22°C and a humidity of 70%), under standard conditions of controlled light (12/12 h) and granted free access to food and water. Next, 72 MCAO-operated mice were anesthetized via intraperitoneal injection of 1% sodium pentobarbital (P3761; Sigma–Aldrich, St. Louis, MO, United States). Next, the scalp of mice was cut open with the skull subsequently exposed and connected to cerebral blood flow monitor. A 1-cm long median longitudinal incision was made from the mandible to sternal stalk in an attempt to identify the left carotid sheath, the common carotid artery, external carotid artery, as well as the internal carotid artery. The proximal end of common carotid artery was double ligated, the external carotid artery was separated and ligated, after which the internal carotid artery was separated. The internal carotid artery was clipped with a micro-arterial clip, with a small opening made into the wall of external carotid artery. The head end plugged with suture (0.23 mm in diameter of head end and 0.18 mm in diameter of trunk) was inserted into common carotid artery, after which 5/0 suture was employed to ligate the opening of the external carotid artery. An arterial clamp was subsequently removed and suture was extended to internal carotid artery, then up to middle cerebral artery (at a depth of 12.0 mm). Later, the blood flow signal using a cerebral blood flow monitor was analyzed (A decline to around 20% was indicator), with the wound then covered using cotton (soaked with 0.9% sodium chloride injection). After 1 h of occlusion, the suture was removed, followed by double-ligation between entrance of external carotid artery suture and bifurcation of internal carotid artery. The suture on common carotid artery was untied and blood flow from common carotid artery to internal carotid artery was restored. The scalp was sutured until cerebral blood flow as depicted by a flow monitor indicated a return to 100%. The remaining 24 mice were sham-operated whose middle cerebral arteries were not inserted with suture. During the operative procedures, body temperature of mice was maintained, while food and water were freely available post-operation.

The experimental models were successfully established in 60 mice reflected by a success rate of 83.33%. A total of 24 mice served as the MCAO mouse models, while the remaining MCAO mice were administered with EVs derived from MSCs co-transfected with mimic-negative control (NC) + oe-NC (EV-mimic-NC + oe-NC), with miR-132-mimic + oe-NC (EV-miR-132 mimic + oe-NC) or with miR-132 mimic + oe-c-jun respectively (*n* = 12). Briefly, corresponding adenovirus (ADV) were injected via the cerebral ventricle 2 h prior to MCAO establishment. Following anesthesia, the mice were placed on a stereotactic head frame (RWD Life Science Co., Ltd., San Diego, CA, United States). A middle line incision was made at the head region after which a hole was drilled into the right side of the skull (0.5 mm posterior and 1.0 mm lateral to bregma). A Hamilton syringe was subsequently used to load and inject 4 μL of ADV into ventricle (2.5 mm vertically) at 0.2 μL/min *via* micro-injection pump (KDS 310; KD Scientific Inc., Holliston, MA, United States), and needle was retained for 5 min after injection to prevent leakage. After the needle was removed, drilled hole was blocked and incision was immediately sutured (Zuo et al., 2019). Lastly, 24 h-post MCAO, 0.2 mL of phosphate-buffered saline (PBS) containing 200 μg EVs was injected through tail vein, whereas the same amount of PBS was injected in 24 MCAO mouse models as controls (Zhang et al., 2019).

### OGD Treatment

One-day-old C57BL/6J mice were placed in HBSS (14170112; Gibco Life Technologies, Grand Island, NY, United States) supplemented with 1 mM sodium pyruvate (11360070; Gibco) and 10 mM HEPES (15630080; Gibco) in the absence of Ca^2+^ and Mg^2+^. The hippocampus was then detached in HBSS containing 0.125% trypsin (25200056; Gibco) at 37°C for 10 min. The hippocampus was then dispersed into a single cell suspension. DMEM containing 10% FBS was then added to terminate detachment (10569044; Gibco). The dispersed hippocampus was then stood for 3 min. The supernatant was subsequently transferred to a new centrifuge tube and centrifuged at 2000 r/min for 2 min. The pellet was then added with B-27 (17504044; Gibco), 0.5 mM L-glutamine (25030081; Gibco), 20 IU/mL penicillin, and 20 IU/mL Streptomycin (15070063; Gibco) in Neurobasal medium (21103049; Gibco). The single cells were seeded at 4 × 10^6^ cells/well into a six-well plate coated with PDL (100 μg/mL) (A3890401; Gibco). The cells were then cultivated at 37°C under humidified atmosphere conditions comprised of 5% CO_2_ in an incubator. Half of glutamate-free fresh medium was replaced every 2–3 days. The purity of neurons was confirmed to be approximately 95%. Follow-up experiments were performed after 7 days of culture.

After the neurons had been infected with corresponding lentivirus for 6 h, medium was replaced with a new medium. After 18–24 h of culture, neurons were exposed to OGD conditions (Xu et al., 2020). In order to simulate neuronal hypoxia-ischemia *in vitro*, neurons were treated with OGD. The original medium was discarded and added with glucose-free DMEM (11966025; Gibco), and incubated in anaerobic chamber with 5% CO_2_ and 95% N_2_ for 4 h, while that of the normal controls were cultured in DMEM (SLM-020-A; Sigma–Aldrich) over a 4 h period. After OGD treatment had been performed, all neurons were switched to culture with Neurobasal medium in a 5% CO_2_ incubator at 37°C for 24 h. Neurons were infected with lentiviruses containing sh-NC, sh-Acvr2b-1, sh-Acvr2b-2, mimic-NC, miR-132 mimic, EV-mimic-NC, EV-miR-132 mimic, EV-mimic-NC + vector, EV-miR-132 mimic + vector, EV-miR-132 mimic + Acvr2b, short hairpin RNA (sh)-NC + oe-NC, sh-Acvr2b + oe-NC, sh-Acvr2b + oe-c- jun, EV-mimic-NC + oe-NC, EV-miR-132 mimic + oe-NC, EV-miR-132 mimic + oe-c-jun. All lentiviruses were purchased from Sangon (Shanghai, China), while the primer sequences and plasmid construction completed by Sangon.

### Neurological Scores

A blinded investigator performed the neurological examination which was scored 24 h after MCAO (Garcia et al., 1995). The scoring system was comprised of seven sub-tests, including spontaneous activity, symmetry in movement of four limbs, forepaw outstretching, climbing, body proprioception, response to vibrissae touch, and beam walking. Neurological scores ranged from 3 (most severe deficits) to 21 (normal).

### Behavioral Testing

Pole test was performed 24 h post operatively. The mice were placed on a vertical wooden pole with 50 cm in length, 8 mm in diameter, and a rough surface. The time taken for mouse to turn completely head downward (T_turn_) and total time taken to descend down and reach floor with its four paws (T_total_) were both recorded.

The foot fault test was also conducted 24 h after operation. Briefly, mice were placed on a horizontal grid floor elevated above surface and allowed to walk for 2 min. A foot fault was recorded in the event of the mouse’s foot miss-stepping on the grid or when the foot fell downward through the openings between the grids. All four limbs were observed for misses. The percentage of total foot faults was recorded and calculated.

### Hematoxylin and Eosin (HE) Staining

Complete hippocampus sections were selected and dried at room temperature. The sections were fixed with paraformaldehyde for 30 s at room temperature and stained with hematoxylin (60°C) for 60 s. Next, differentiation was performed by adding 1% acid alcohol for 3 s, followed by staining with eosin for 3 min. Dehydration was performed using ethanol at a concentration of 70%, 80%, 95%, and absolute ethanol for 5 min, followed by dewaxing with Xylene for 5 min (three times). Finally, the sections were transparently sealed with neutral gum, with the lesions degree evaluated under a microscope (BX63; Olympus Corporation, Japan).

### Terminal Deoxynucleotidyl Transferase-Mediated dUTP Nick End-Labeling (TUNEL) Staining

TUNEL staining was performed using an Apoptosis detection kit (C1098; Beyotime, Shanghai, China). Briefly, the sections were dewaxed with xylene for 5 min (twice), followed by dewaxing using absolute ethanol, 90% ethanol for 2 min, 70% ethanol or distilled water for 5, 2, 2, or 2 min, respectively. Next, 20 μg/mL of DNase-free proteinase K was added in a dropwise manner with the neurons then treated at 37°C for 15 min. Next, 50 μL of TUNEL detection solution was added to the sample and incubated at 37°C for 60 min under conditions void of light. Finally, the sections were mounted using 4’,6-diamidine-2-phenylindole (DAPI) with cell apoptosis evalutated under a fluorescence microscope (BX63; Olympus).

### 2,3,5-Triphenyltetrazolium Hydrochloride (TTC) Staining

One week post modeling, the mice brains were collected, sectioned coronally at 1 mm interval, and stained with TTC at 37°C for 20 min. The brain sections were then fixed in 2% paraformaldehyde and photographed with a digital camera (IXUS175, Canon, Tokyo, Japan). The infarcted area exhibited white and non-infarcted area was pink. ImageJ software was utilized to quantify infarcted area of section. The infarct volume was calculated based on previous research methods (Zuo et al., 2019).

### Isolation and Cultivation of BMSCs

C57BL/6J mice were administered with an intraperitoneal injection of 3% sodium pentobarbital. Bone marrow was collected from femurs and tibias under aseptic conditions. Proximal and distal bones were collected with the bone marrow cavity exposed. The bone marrow was repeatedly flushed with 6 mL of DMEM and centrifuged. The collected bone marrow was suspended in DMEM/F12 (1:1) (11330032; Gibco), supplemented with 10% FBS (10099141; Gibco) and 1% penicillin-streptomycin (15070063; Gibco). The suspension was subsequently cultured under 5% CO_2_ incubator at 37°C for 48 h. The medium was replaced every 2–3 days in order to discard any non-adherent cells and debris. When the cells reached 70–80% confluence, 0.25% trypsin (SM-2001; Sigma–Aldrich) was added followed by cell passage on three separate occasions. BMSCs were identified using flow cytometry with CD29 (12-0291-82; Thermo Fisher Scientific, Waltham, MA, United States), CD44 (14-0441-82; Thermo Fisher Scientific), SCA-1 (17- 5981-82; Thermo Fisher Scientific), CD31 (11-0311-82; Thermo Fisher Scientific), FLK-1 (13-5821-82; Thermo Fisher Scientific), and CD45 (14-0451-82; Thermo Fisher Scientific). Alizarin red staining (ST1078; Beyotime) was applied to detect osteogenic differentiation ability of BMSCs. Oil Red O staining (E607319; Sangon) was employed to determine the adipogenic differentiation ability of BMSCs. Alcian Blue staining was employed to measure chondrogenic differentiation of BMSCs (A600298; Sangon) (Lin et al., 2018). BMSCs were either transfected with plasmids containing mimic-NC or miR-132 mimic.

### Isolation and Identification of EVs

The BMSC medium was replaced with serum-free medium. BMSCs were centrifuged at 300 *g* for 10 min, followed by another 2000 *g* for another 10 min. The BMSCs medium supernatant was then harvested and ultra-centrifuged at 100,000 *g* for 1 h at 4°C. The precipitate was resuspended with 200 μL PBS and stored at −80°C.

A TEM was adopted to identify structure of EVs. Briefly, 30 μL of EVs was added in a dropwise manner on a copper wire. After standing for 1 min, EVs were blotted from side with filter paper, 30 μL of phosphotungstic acid solution (pH 6.8) was added dropwise, and counter-stained at room temperature for 5 min. The EVs were dried via exposure to incandescent light bulb, followed by observation under a Tecnai 10 TEM (FEI Company, Hillsboro, OR, United States). The EV-related protein levels including CD9, CD63, and TSG101 and endoplasmic reticulum-derived protein CANX were detected by western blot analysis.

Dynamic light scattering was employed to determine the diameter of the EVs. Briefly, Zetasizer Nano-ZS90 (Malvern Panalytical, Malvern, United Kingdom) was applied to excite the light wavelength (λ = 532 nm). EV samples were diluted with 0.15 M NaCl to appropriate level of optical signal detection (the ratio was 1: 50). The particles were illuminated by the laser, with their movement captured for 60 s. The recorded movements were analyzed using Nanosight particle tracking software [Version nanoparticle tracking analysis (NTA) 3.1] to calculate EV the concentrations and size distribution. Particles with diameters between 30 and 120 nm were considered successfully isolated EVs, with the EVs applied at a concentration of 10 μg/mL for 24 h (Koritzinsky et al., 2017). PKH67 (Green)-labeled EVs were co-cultured with neurons and uptake of EVs by neurons was observed under a confocal fluorescence microscope (FV1000, Olympus).

### Reverse Transcription-Quantitative Polymerase Chain Reaction (RT-qPCR)

The total tissue or RNA was extracted using Trizol (16096020; Thermo Fisher Scientific). The miRs were synthesized using a miRNA first-strand cDNA (tailing method) kit (B532451; Sangon), whereas mRNA was synthesized using cDNA first-strand synthesis kit (D7168L; Beyotime) in accordance with the manufacturer’s instructions. RT-qPCR experiments were performed using an RT-qPCR kit (Q511-02; Vazyme Biotech Co., Ltd., Nanjing, China) in line with manufacturer’s protocol. PCR amplification was performed using a Bio-Rad real-time PCR instrument CFX96. U6 was employed as a miR-132 internal reference while GAPDH was regarded as an internal reference for Acvr2b. The U6 primers and universal downstream primers were provided by miR reverse transcription kit. The primer sequences of miR-132, Acvr2b, and GAPDH were designed and provided by Sangon. The primer sequences are depicted in Table 1. ΔΔCt = ΔCt experimental group −ΔCt control group, where ΔCt = Ct _(target gene)_ − Ct _(internal reference)._

**TABLE 1:** RT-qPCR primer sequences.

Gene	Primer sequences (5’-3’)
miR-132	F: TAACAGTCTACAGCCATGGTCG
Acvr2b	F: ACCCCCAGGTGTACTTCTG
	R: CATGGCCGTAGGGAGGTTTC
GAPDH	F: AGGTCGGTGTGAACGGATTTG
	R: TGTAGACCATGTAGTTGAGGTCA

*Note: MiR-132, microRNA-132; Acvr2b, Activin receptor type IIB; GAPDH, glyceraldehyde-phosphate dehydrogenase; F, forward; R, reverse.*

### Western Blot Analysis

A radioimmunoprecipitation assay containing phenylmethylsulfonyl fluoride (P0013B; Beyotime) was added to the tissue and cell lysate for total protein extraction. The nuclear and cytoplasmic protein was extracted using kit (P0028; Beyotime) as per the manufacturer’s instructions. Next, 8–12% sodium dodecyl sulfate gel was used for gel-electrophoretic separation. The proteins on gel were then electro-transferred onto a polyvinylidene fluoride membrane (1620177; Bio-Rad, Hercules, CA, United States). The membrane was then blocked with either 5% skim milk or 5% bovine serum albumin at room temperature for 1 h, followed by the introduction of primary rabbit anti-mouse monoclonal antibodies (Abcam, Cambridge, United Kingdom) against GAPDH (ab181602; 1: 5000), Acvr2b (ab180185; 1: 1000), Smad2 (ab63576; 1: 1000), p-Smad2 (ab53100; 1: 1000), c-jun (ab31419; 1: 1000), CD9 (ab92796; 1: 1000), CD63 (ab217345; 1: 1000), Tumor Susceptibility Gene 101 (TSG101; ab125011; 1: 1000), CANX (ab22595; 1: 1000), B-cell lymphoma-2 (Bcl-2; ab182858; 1: 1000), Bcl-2-Associated X (Bax; ab32503; 1:1000), Cleaved-Caspase 3 (ab49822; 1:1000), and Caspase 3 (ab13847; 1:1000) for overnight incubation at 4°C. The following day, tge membrane was washed three times with TBST for 1 min at room temperature (5 min per wash). Horseradish peroxidase-labeled goat anti-rabbit Immunoglobulin G secondary antibody (ab6721; 1:5000, Abcam) was then added dropwise to membrane and incubated at room temperature for 1 h. Under room temperature conditions, the membrane was washed three tines with TBST, each time for 5 min. Next, the membrane was immersed in electrochemiluminescence reaction solution (1705062; Bio-Rad) at room temperature for 1 min. The membrane was then subjected to an Image Quant LAS 4000C gel imager (General Electric Company, Boston, MA, United States). The total protein of the cells was determined using GAPDH as an internal reference, with the ratio of gray value of target band to internal reference band was used as relative expression level of protein. The expression level of each protein was detected.

### Cell Counting Kit-8 (CCK-8) Assay

After the neurons were treated with OGD and recovered, cell proliferation was determined by using CCK-8 kit (GK10001; GLPBIO, Montclair, CA, United States) based on the manufacturer’s instructions. Briefly, 20 μL of CCK-8 reagent was added to each well, with the neurons mixed and incubated in cell incubator for 4 h. Cell viability (%) was calculated based on the following formula: (A_450_ of test well/A_450_ of control well) × 100. A cell viability histogram was subsequently plotted based on the data obtained.

### Flow Cytometry

Apoptosis was detected using Annexin V-FITC and PI kit (C1062L; Beyotime). Neurons were treated, cultured in an incubator for 48 h, and collected in a 200 μL buffer, followed by supplementation with 10 μL Annexin V-FITC and 5 μL PI for 15 min at room temperature under conditions void of light. Next, 300 μL buffer was added followed by the use of flow cytometry (Becton, Dickinson and Company, Franklin Lake, NJ, United States) to detect cell apoptosis and subsequently calculate the rate of apoptosis.

### Lactate Dehydrogenase (LDH) Secretion

The LDH leakage was performed to evaluate neuronal damage using an LDH-cytotoxicity assay kit (CYTODET-RO, Sigma–Aldrich) in strict adherence with the manufacturer’s instructions. The neuronal medium was collected following OGD treatment, neurons were then centrifuged to obtain supernatant and lyzed with 1% Triton X-100 (T9284, Sigma–Aldrich), followed by centrifugation to remove cellular debris and cell lysate was obtained. The cell supernatant and cell lysate were incubated with LDH reaction mixture at 37°C for 15 min. The absorbance was read at 490 nm using a microplate reader when the reaction was terminated, and LDH release was expressed as a percentage of total LDH.

### Dual-Luciferase Reporter Gene Assay

An online analysis website http://mirdb.org/was explored to predict possible binding site between miR-132 and Acvr2b. A Dual-luciferase reporter gene assay was subsequently conducted to verify their targeting relationship. Acvr2b mRNA 3’ UTR gene fragment [Acvr2b wild type (Wt)] and mutant fragment [Acvr2b mutant type (Mut)] targeted to miR-132 were synthesized, introduced into pGL3-basic vector (E1751; Promega Corporation, Madison, WI, United States), and digested with restriction enzyme T4 DNA ligase (M0204S, New England, Biolabs, Beverly, MA, United States) in order to insert a target fragment into pGL3 vector. Renilla fluorescent plasmid and constructed luciferase reporter plasmid transfected with mimic-NC and miR-132 mimic were then co-transfected into HEK293T cells, respectively. The transfected HEK293T cells were placed into a 5% CO_2_ and 37°C saturated humidity incubator for further cultivation. The cells were then collected and lyzed 48 h after transfection. The Dual-Luciferase^®^ Repeater Assay System Kit (E1910, Promega Corporation) was employed in GloMax^®^ 20/20 Luminometer (E5311, Promega Corporation) for luciferase activity. All vectors were constructed by Sangon.

### Immunohistochemistry

Tissue sections were dewaxed and rehydrated with xylene and graded alcohol, followed by incubation in a citrate solution for 10 min for antigen retrieval. The tissue sections were subsequently blocked with 1% bovine serum albumin for 2 h at room temperature and incubated with rabbit anti-mouse Acvr2b antibody (ab128544; 1: 100, Abcam) at 4°C overnight, followed by incubation with goat anti-rabbit secondary antibody (ab6721; 1: 500, Abcam) at room temperature for 45 min. Finally, the sections were developed using a DAB kit (P0203, Beyotime, Shanghai), and analyzed under a microscope (BX63, Olympus, Japan).

### Statistical Analysis

All statistical analyses were performed using SPSS 21.0 (IBM, Armonk, NY, United States). Measurement data were expressed as the mean ± standard deviation based on the results of three independent tests. An unpaired *t*-test was conducted to compare data between two groups. One-way ANOVA was conducted for multiple group comparisons, followed by the application of a Tukey’s *post hoc* test. Statistical significance was reflected by *p* < 0.05.

## Results

### Acvr2b Expression Was Highly Expressed in MCAO Mice and OGD-Treated Neurons

**FIGURE 1:**
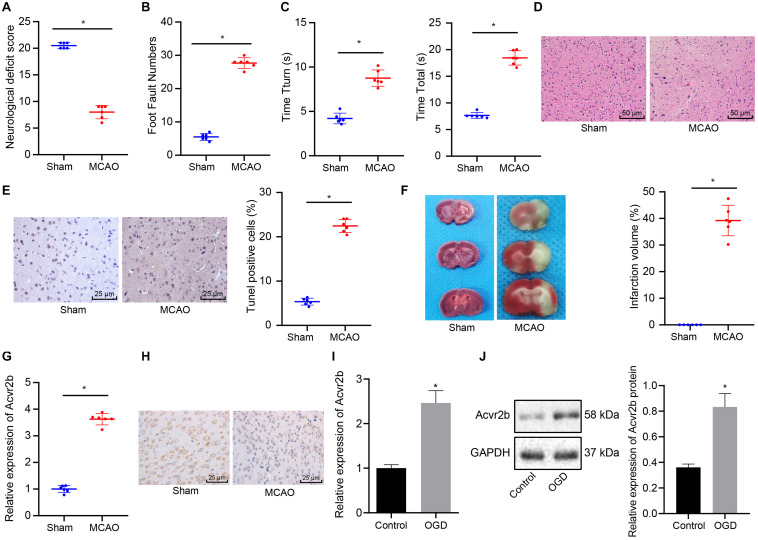
Upregulation of Acvr2b expression was observed in MCAO mice and OGD-treated neurons. **(A)** Neurological score was evaluated in sham-operated mice and MCAO mice (*n* = 6/group). **(B)** The number of mouse’s foot miss-steps measured using foot fault test. **(C)** Time taken for mice to turn 180° headfirst (T_turn_) and to reach ground detected by pole test. **(D)** Pathological changes of hippocampal tissues were observed using HE staining (200×). **(E)** Apoptotic rate of neurons examined using TUNEL staining (400×). **(F)** Infarct volume detected using TTC staining. **(G)** The expression of Acvr2b examined using RT-qPCR. **(H)** The expression of Acvr2b in mice detected using IHC staining (400×). **(I)** The mRNA expression of Acvr2b in OGD-treated neurons measured by RT-qPCR. **(J)** The protein expression of Acvr2b in OGD-treated neurons measured using western blot analysis normalized to GAPDH. *p < 0.05 *vs.* sham-operated mice or normal controls. The data between two groups were compared using unpaired *t*-test.

Existing literature has indicated that Acvr2b is highly expressed in neonatal HIE neuron injury while highlighting its correlation to HIE severity (Looney et al., 2017). To further verify the role of Acvr2b in ischemic neuron injury, a MCAO mouse model and OGD neuron model were constructed. The neurological score results provided evidence of the MCAO mice neurological score 24 h post-surgery was markedly decreased when compared to sham-operated mice (Figure 1A). The foot fault test revealed that number of stomping on right injured limb in MCAO mice was increased when compared to sham-operated mice (Figure 1B). The Pole test results provided revealed that the time taken for MCAO mice to turn 180° headfirst (T_turn_) and to reach ground (T_total_) were significantly longer on day 1 and 3 relative to the sham-operated mice (Figure 1C). HE staining exhibited that pathological changes in MCAO mice was more distinct than those in sham-operated mice (Figure 1D). TUNEL staining displayed that apoptotic rate of neurons in MCAO mice was strikingly higher compared to that of sham-operated mice (Figure 1E). TTC staining analyzed that infarct volume in MCAO mice was dramatically raised when compared to that observed in sham-operated mice (Figure 1F). The aforementioned results provided verification of the successful establishment of MCAO mouse model. RT-qPCR and immunohistochemistry methods demonstrated that Acvr2b expression was remarkably upregulated in MCAO mice in comparison with that in sham-operated mice (Figures 1G,H). RT-qPCR and western blot analysis displayed that Acvr2b expression was elevated in neurons exposed to OGD conditions (Figures 1I,J). Taken together, the results obtained indicated that Acvr2b was highly expression expressed in MCAO mice and OGD-exposed neurons.

### Silencing of Acvr2b Has a Protective Effect on Neuronal Injury

**FIGURE 2:**
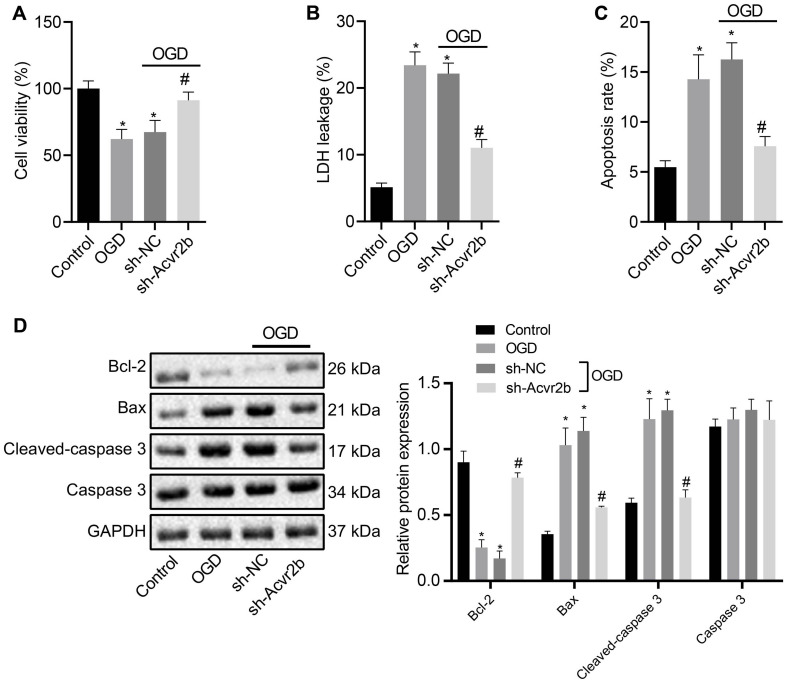
Silencing of Acvr2b exerts a protective effect on neuronal injury. **(A)** Neuronal viability after silencing of Acvr2b assessed by CCK-8. **(B)** LDH leakage after silencing of Acvr2b detected by LDH secretion. **(C)** Apoptotic rate after silencing of Acvr2b evaluated using flow cytometry. **(D)** The expression of Bcl-2, Bax, and Cleaved-caspase 3 and protein expression of Caspase 3 after silencing of Acvr2b determined by western blot analysis normalized to GAPDH. *p < 0.05 *vs.* normal controls. #*p* < 0.05 *vs.* sh-NC transfected neurons. An unpaired *t*-test was performed for comparisons of data between two groups. One-way ANOVA was conducted for multiple group comparisons, followed by Tukey’s *post hoc* test.

Next, to further elucidate the effect of Acvr2b on neuronal apoptosis, RT-qPCR and western blot analyses were performed to assess the silencing efficiency of Acvr2b in OGD-exposed neurons. The results obtained revealed that expression of Acvr2b in neurons transfected with sh-Acvr2b-1 and sh-Acvr2b-2 was lower than that in sh-NC transfected neurons (Supplementary Figures S1A,B). sh-Acvr2b-1 exhibited the highest silencing efficiency in the neurons and was selected for subsequent experiments. The CCK-8 results provided evidence attesting that the neuronal viability was significantly decreased in both OGD-treated neurons. However, neurons transfected with sh-Acvr2b displayed enhanced neuronal activity (Figure 2A). LDH leakage was detected via LDH secretion, with the results demonstrating that the OGD-treated neurons had a greater degree of LDH release. Additionally, LDH leakage in sh-Acvr2b transfected neurons was lowered (Figure 2B). The flow cytometry results revealed that the OGD-treated neurons exhibibted an augmented apoptotic rate. Moreover, apoptotic rate in sh-Acvr2b transfected neurons was markedly decreased (Figure 2C). The western blot analysis results of revealed that Bcl-2 expression in sh-Acvr2b transfected neurons was elevated, while Bax and Cleaved-caspase 3 expression was markedly declined, whereas Caspase 3 expression remained unchanged (Figure 2D). The aforementioned results revealed that silencing of Acvr2b could confer protection against neuron injury.

### MiR-132 Targets and Inhibits Expression of Acvr2b

**FIGURE 3:**
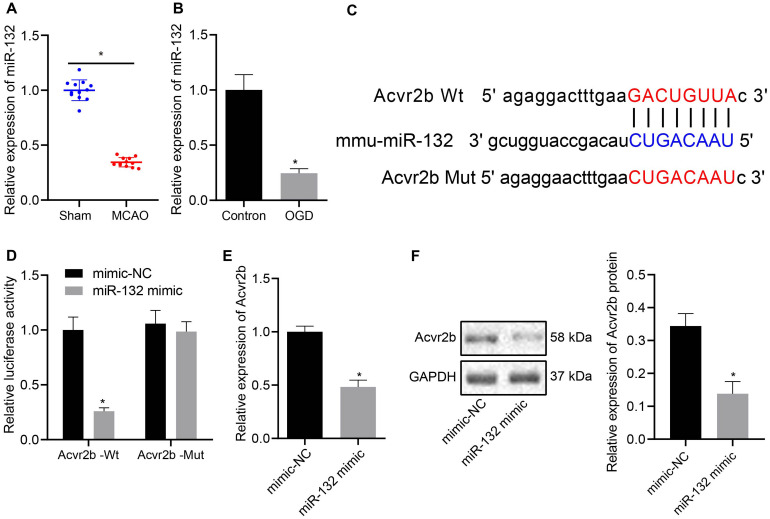
MiR-132 targets and represses Acvr2b expression. **(A)** miR-132 expression in MCAO mice measured using RT-qPCR. **(B)** The expression of miR-132 in neurons detected using RT-qPCR. **(C)** The targeting relationship between miR-132 and Acvr2b mRNA at 3 ‘UTR predicted by online website (http://mirdb.org/). **(D)** The targeting relationship between miR-132 and Acvr2b verified by using dual-luciferase reporter gene assay. **(E)** Acvr2b mRNA expression after overexpressing miR-132 expression examined using RT-qPCR. **(F)** The protein expression of Acvr2b after overexpressing miR-132 expression determined by western blot analysis normalized to GAPDH. *p < 0.05 *vs.* sham-operated mice (*n* = 6/group), normal controls or mimic-NC transfected HEK293T cells. An unpaired *t*-test was performed for comparisons of data between two groups.

Prior studies have proposed the notion that miR-132 confers neuronal protection against OGD-induced apoptosis and its upregulation resulted in attenuated cerebral injury in MCAO mice (Sun et al., 2017; Zuo et al., 2019). In this study, RT-qPCR results demonstrated that miR-132 expression in MCAO mice was notably downregulated when compared to that in sham-operated mice (Figure 3A), whule the expression of miR-132 was notably decreased in OGD-treated neurons when compared to that in normal controls (Figure 3B). In subsequent experiments, online analysis site http://mirdb.org/ was adopted to predict targeted binding site of miR-132 to 3’UTR of Acvr2b mRNA (Figure 3C). The RT-qPCR results revealed that miR-132 expression in miR-132 mimic transfected neurons and HEK293T cells was elevated when compared with mimic-NC transfected neurons and HEK293T cells (Supplementary Figures S2A,B). Dual-luciferase reporter gene assay results provided evidence indicating that HEK293T cells co-transfected with miR-132 mimic and Acvr2b Wt induced decreased fluorescence activity in comparison with that co-transfected with mimic-NC and Acvr2b Wt, while replacement with Acvr2b Mut transfected cells failed to exhibit a significant difference (Figure 3D). The RT-qPCR and western blot analysis results revealed that Acvr2b expression in HEK293T cells transfected with miR-132-mimic was markedly declined (Figures 3E,F). The aforementioned results suggested that miR-132 could target and repress Acvr2b expression.

### EV-Derived miR-132 Released From MSCs Mitigates Neuronal Injury by Targeting and Suppressing Acvr2b Expression

**FIGURE 4:**
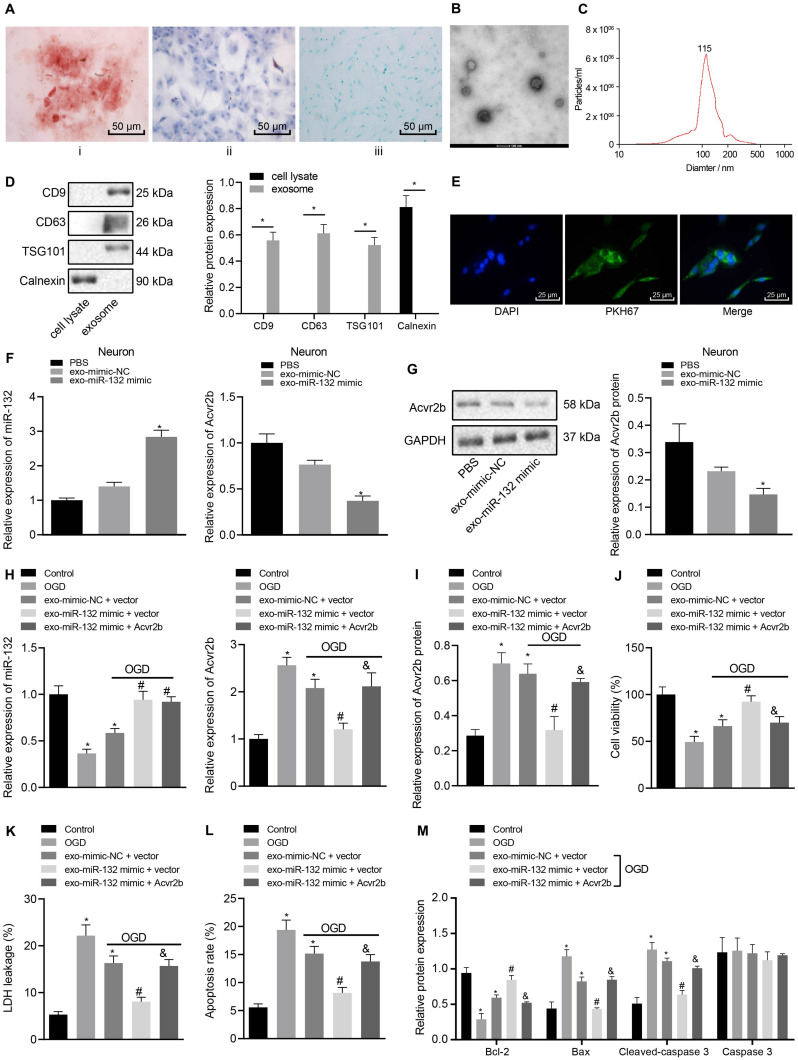
EV-derived miR-132 from MSCs attenuates neuron injury by targeting and repressing Acvr2b expression. **(A)** BMSCs directed differentiation was detected (200×). **(B)** Structural diagram of EVs observed by transmission electron microscopy (100 nm). **(C)** The size of EVs measured using dynamic light scattering. **(D)** EV-related protein bands determined by western blot analysis normalized to GAPDH. **(E)** The uptake of EVs by neurons observed under confocal fluorescence microscopy (400×). **(F)** The expression of miR-132 and Acvr2b in neurons detected by RT-qPCR. **(G)** The protein expression of Acvr2b in neurons detected by western blot analysis normalized to GAPDH. **(H)** The expression of miR-132 and Acvr2b in neurons after different treatments detected by RT-qPCR. **(I)** The protein expression of Acvr2b after different treatments detected by western blot analysis normalized to GAPDH. **(J)** Neuron activity after different treatments detected by CCK-8. **(K)** LDH leakage rate of neuron detected by LDH after different treatments. **(L)** The apoptosis rate of neurons after different treatments detected using flow cytometry. **(M)** The expression of bcl-2, Bax, Cleaved caspase 3, and caspase 3 in neurons after different treatments measured by western blot analysis normalized to GAPDH. *p < 0.05 *vs.* EV-mimic-NC treated neurons or normal controls. #*p* < 0.05 *vs.* EV-mimic-NC + vector co-transfected neurons. &*p* < 0.05 *vs.* EV-miR-132 mimic + vector co-transfected neurons. An unpaired *t*-test was performed for comparisons of data between two groups. One-way ANOVA was conducted for multiple group comparisons, followed by Tukey’s *post hoc* test.

Bone MSCs-released EVs were proposed to alleviate ischemic neuron injury and were found abundant in miR-132 (Ma et al., 2018; Xiao et al., 2018). Hence, we speculated that miR-132 may be derived from MSCs-released EVs and target Acvr2b. In order to evaluate this hypothesis, we identified BMSCs isolated from mice *in vitro*. The flow cytometry results indicated that CD29 (97.12%), CD44 (99.03%), and SCA-1(96.18%) were highly expressed while CD31 (5.27%), FLK-1 (4.86%), and CD45 (5.48%) were poorly expressed. Besides, results indicating the directional differentiation ability of the BMSCs revealed that cultured cells were capable of osteogenic, lipogenic and chondrogenic differentiation (Figure 4A), suggesting that the BMSCs had been successfully isolated. Transmission electron microscopy revealed a clear membrane bound on EVs (Figure 4B). EVs were detected to be varied in size from 30 to 120 nm by DLS (Figure 4C). The protein expression of the EV-related proteins CD9, CD63, TSG101, and endoplasmic reticulum calcium-binding protein (CANX) were determined via western blot analysis, the results of which demonstrated that CD9, CD63, and TSG101 were highly expressed while CANX was not expressed in EVs (Figure 4D). These results indicated that the EVs had been successfully extracted from the MSCs. Confocal fluorescence microscopy exhibited that uptake of PKH67-EVs by neurons was distinct 48 h after co-culture (Figure 4E), suggesting that EVs could be transferred to neuronal cells.

The RT-qPCR results revealed that expression of miR-132 in miR-132 mimic-transfected BMSCs was notably elevated (Supplementary Figure S3A). The expression of miR-132 in EV-miR-132 mimic-transfected BMSCs was also increased (Supplementary Figure S3B). The RT-qPCR results demonstrated that miR-132 expression in EV-miR-132 mimic-transfected neurons strikingly rose, but expression of Acvr2b was downregulated (Figure 4F). Western blot analysis results revealed that Acvr2b protein expression in neurons transfected with EV-miR-132 mimic was remarkably declined (Figure 4G).

To further elucidate the effect of EV-derived miR-132-Acvr2b axis on neuron injury, expressions of miR-132 and Acvr2b in neurons following different treatments were determined by RT-qPCR and western blot analysis. The results suggested that the expression of miR-132 was markedly increased in neurons by EV-miR-132 mimic. Besides, the expression of miR-132 in the neurons transfected with EV-miR-132 mimic + vector or EV-miR-132 mimic + Acvr2b was boosted. In addition, the expression of Acvr2b in neurons transfected with EV-mimic-NC + vector displayed significant increases. The expression of Acvr2b in neurons transfected with EV-miR-132 mimic + vector was strikingly. Acvr2b expression in the neurons co-transfected with EV-miR-132 mimic + Acvr2b showed significant elevation when compared to that in neurons co-transfected with EV-miR-132 mimic + vector (Figures 4H,I). The CCK-8 results revealed that neuronal activity in neurons transfected with EV-mimic-NC + vector was decreased compared with that in normal controls. However, the co-transfection of EV-miR-132 mimic + vector leads to enhanced neuronal viability. More intriguingly, neuronal viability in neurons co-transfected with EV-miR-132 mimic + Acvr2b was diminished when compared to the neurons co-transfected with EV-miR-132 mimic + vector (Figure 4J). The LDH results revealed that LDH leakage markedly increased in the neurons transfected with EV-mimic-NC + vector compared with normal controls, whereas LDH leakage in neurons transfected with EV-miR-132 mimic + vector was dramatically decreased. Co-transfection of EV-miR-132 mimic + Acvr2b in neurons induced highly elevated LDH leakage compared with that co-transfection of EV-miR-132 mimic + vector (Figure 4K). The flow cytometry results revealed that neuronal apoptosis in neurons transfected with EV-mimic-NC + vector was markedly elevated in comparison with that in normal controls. However, co-transfection of EV-miR-132 mimic + vector showed decreased apoptosis. Neuronal apoptosis in neurons co-transfected with EV-miR-132 mimic + Acvr2b was elevated in comparison with that co-transfected with EV-miR-132 mimic + vector (Figure 4L). The western blot analysis results revealed that Bcl-2 expression in neurons transfected with EV-mimic-NC + vector was notably decreased but Bax and Cleaved-caspase 3 expression rose compared with normal controls. The Bcl-2 expression in the neurons transfected with EV-miR-132 mimic + vector was increased, whereas expression of Bax and Cleaved-caspase 3 was reduced. Besides, expression of Bcl-2 in neurons transfected with EV-miR-132 mimic + Acvr2b was lower than that of EV-miR-132 mimic + vector (*p* < 0.05), while the expression of Bax and Cleaved-caspase 3 was increased, and there was no significant difference in Caspase 3 expression (*p* > 0.05; Figure 4M). The aforementioned results suggested that EV-derived miR-132 from MSCs could attenuate neuron injury by targeting and repressing Acvr2b expression.

### Acvr2b Activates p-Smad2/c-jun Signaling Pathway, Hence Inducing Neuronal Injury

**FIGURE 5:**
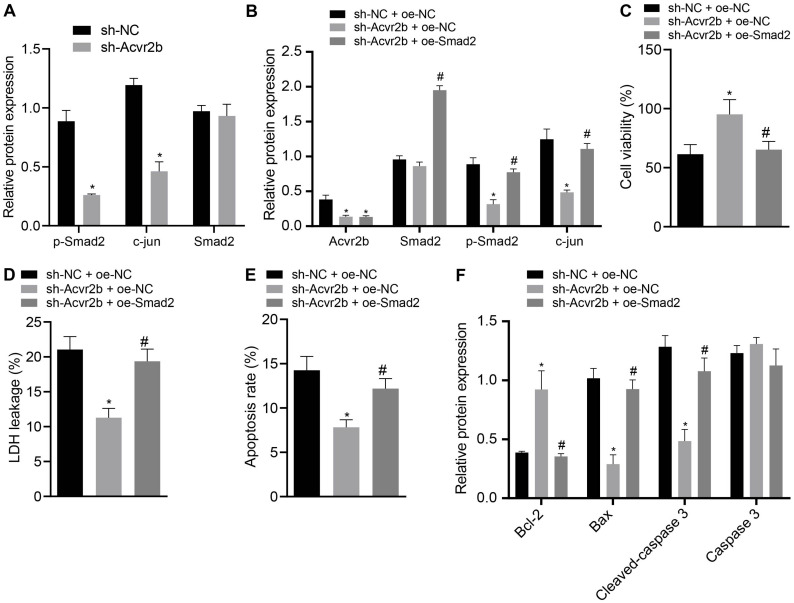
Acvr2b activates Smad2/c-jun signaling pathway, thus inducing neuronal injury. **(A)** The expression bands and statistical histogram of Smad2, p-smad2, and c-jun in neurons after Acvr2b silencing detected by western blot analysis normalized to GAPDH. **(B)** The expression bands and statistical histograms of Acvr2b, Smad2, p-Smad2, and c-jun in neurons after different treatments measured using western blot analysis normalized to GAPDH. **(C)** Statistical histograms of neuron activity after different treatments detected by CCK-8. **(D)** Statistical histogram of neuron LDH leakage rate after different treatments detected by LDH kit. **(E)** The apoptosis rate of neurons after different treatments evaluated using flow cytometry. **(F)** The expression of Bcl-2, Bax, Cleaved caspase 3, and caspase 3 in neurons after different treatments detected by western blot analysis normalized to GAPDH. *p < 0.05 *vs.* sh-NC transfected neurons or sh-NC + oe-NC co-transfected neurons. #*p* < 0.05 *vs.* sh-Acvr2b + oe-NC co-transfected neurons. An unpaired *t*-test was performed for comparisons of data between two groups. One-way ANOVA was conducted for multiple group comparisons, followed by Tukey’s *post hoc* test.

The Acvr2b silencing efficiency following silencing of its expression was initially detected. The western blot analysis results indicated that the expression of p-Smad2 and c-jun in neurons transfected with sh-Acvr2b was decreased, but expression of Smad2 exhibited no distinct difference (Figure 5A).

In order to further elucidate that Acvr2b-induced neuron injury was achieved through the Smad2/c-jun signaling pathway, western blot analysis indicated that Acvr2b expression in neurons transfected with sh-Acvr2b + oe-NC + sh-Acvr2b or sh-Acvr2b + oe-Smad2 was downregulated. Compared with that in neurons transfected with sh-NC + oe-NC, there was no significant difference in Smad2 in neurons transfected with sh-Acvr2b + oe-NC, while neurons transfected with sh-Acvr2b + oe-Smad2 showed highly increased Smad2 expression, compared with that in neurons transfected with sh-Acvr2b + oe-NC. Expression of c-jun in neurons transfected with sh-Acvr2b + oe-NC was reduced. However, the expression of c-jun among the neurons transfected with sh-Acvr2b + oe-Smad2 was elevated when compared to that transfected with sh-acvr2b + oe-NC (Figure 5B).

The CCK-8 results revealed that neuronal activity in the neurons transfected with sh-Acvr2b + oe-NC was enhanced, which was neutralized by the co-transfection of sh-Acvr2b + oe-Smad2 in neurons (Figure 5C). The LDH secretion results suggested that LDH leakage in neurons transfected with sh-Acvr2b + oe-NC was markedly loweed, which was normalized by sh-Acvr2b + oe-Smad2 (Figure 5D). The flow cytometry results revealed that neuronal apoptosis was markedly diminished by silencing Acvr2b, which was annulled by transfection of sh-Acvr2b + oe-Smad2 (Figure 5E). Western blot analysis results indicated that expression of Bcl-2 in neurons was increased, and expression of Bax and Cleaved-caspase 3 was reduced by sh-Acvr2b, which was reversed by sh-Acvr2b + oe-Smad2. There was no significant difference in Caspase 3 expression (Figure 5F). The results obtained provided evidence suggesting that Acvr2b could activate Smad2/c-jun signaling pathway and thereby induce neuron injury.

### Overexpression of c-jun Inhibits Protective Role of EV-miR-132 From MSCs in Neuronal Injury

**FIGURE 6:**
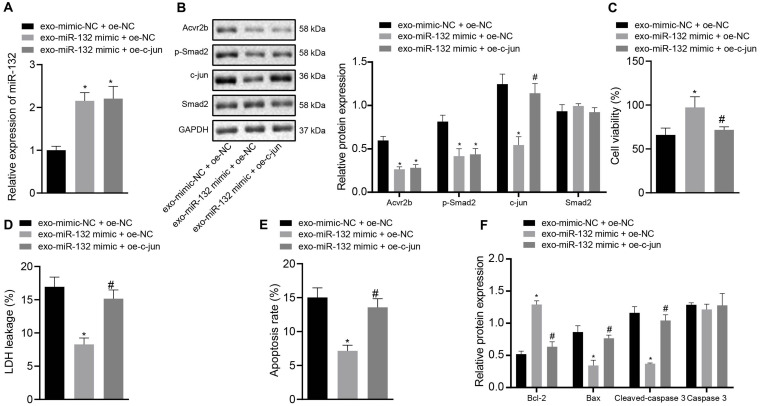
Elevation of c-jun inhibits protective effect of EV-derived miR-132 from MSCs on neurons. **(A)** The expression of miR-132 in neurons detected by RT-qPCR. **(B)** The protein expression levels of Acvr2b, Smad2, p-smad2, and c-jun detected by western blot analysis normalized to GAPDH. **(C)** Neuronal activity after different treatments examined by CCK-8. **(D)** The leakage rate of neuron LDH after different treatments detected using LDH secretion. **(E)** The apoptosis rate of neurons after different treatments detected using flow cytometry. **(F)** The protein expression of Bcl-2, Bax, Cleaved caspase 3, and caspase 3 detected by western blot analysis normalized to GAPDH. *p < 0.05 *vs.* EV-mimic-NC + oe-NC co-transfected neurons. #*p* < 0.05 *vs.* EV-miR-132 mimic + oe-NC co-transfected neurons. An unpaired *t*-test was performed for comparisons of data between two groups. One-way ANOVA was conducted for multiple group comparisons, followed by Tukey’s *post hoc* test.

Next, to ascertain whether EV-derived miR-132 from MSCs exerts protective effects on neurons by inhibiting p-smad2/c-jun signaling pathway, the expression of miR-132 in neurons was determined by RT-qPCR. The protein expression of Acvr2b, Smad2, p-smad2, and c-jun was detected by western blot analysis with the results demonstrating that the expression of miR-132 in neurons co-transfected with EV-miR-132 mimic + oe-NC and EV-miR-132 mimic + oe-c-jun was elevated, while the levels of Acvr2b and p-smad2 were decreased. c-jun in the neurons transfected with EV-miR-132 mimic + oe-NC was markedly downregulated, which was counteracted by EV-miR-132 mimic + oe-c-jun (Figures 6A,B).

The CCK-8 results showed that neuronal activity was notably enhanced by EV-miR-132 mimic, which was abrogated by EV-miR-132 mimic + oe-c-jun (Figure 6C). The results of LDH demonstrating that LDH leakage in neurons transfected with EV-miR-132 mimic was downregulated, which was negated by transfection of EV-miR-132 mimic + oe-c-jun (Figure 6D). Flow cytometry revealed that neuronal apoptosis was declined by EV-miR-132 mimic treatment, while transfection of EV-miR-132 mimic + oe-c-jun reversed this results (Figure 6E). The western blot analysis results demonstrated that expression of Bcl-2 in neurons transfected with EV-miR-132 mimic was increased, whereas expression of Bax and Cleaved-caspase 3 was diminished, which was annulled by transfection of EV-miR-132 mimic + oe-c-jun. Caspase 3 expression exhibited no significant difference (Figure 6F). The aforementioned results suggested that overexpression of c-jun could inhibit protective effect of EV-derived miR-132 from MSCs on neurons.

### EV-Derived miR-132 From MSCs Improves Ischemic Neuronal Injury in MCAO Mice by Blocking p-smad2/c-jun Signaling Pathway *in vivo*

**FIGURE 7:**
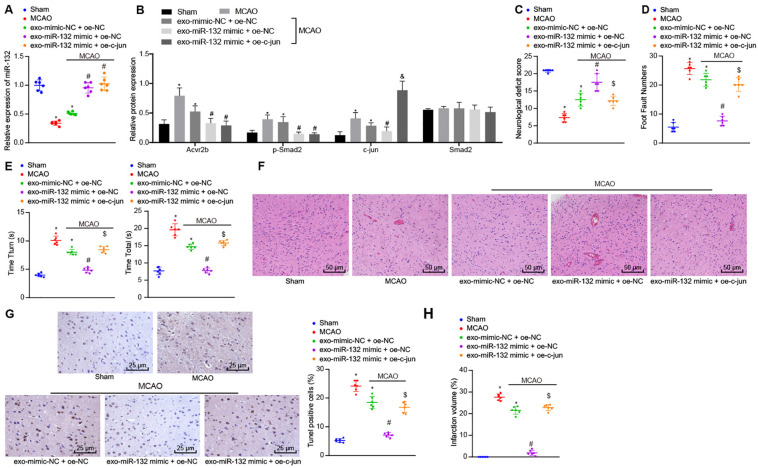
EV-derived miR-132 from MSCs attenuates ischemic neuron injury in MCAO mice by suppressing p-Smad2/c-jun signaling pathway. **(A)** The expression of miR-132 in mice detected by RT-qPCR. **(B)** Protein expression of Acvr2b, Smad2, p-smad2, and c-jun detected by western blot analysis normalized to GAPDH. **(C)** Neurological scores in mice. **(D)** The number of stomping of right injured limb in mice examined by foot fault test. **(E)** The time taken for mice to turn 180° head down (T_turn_) and to reach ground (T_total_) detected using pole test. **(F)** Pathological features in tissues of mice observed using HE staining (200×). **(G)** The apoptosis rate of neurons in hippocampus detected by TUNEL (400×). **(H)** The volume of cerebral infarction measured by TTC staining. *p < 0.05 *vs.* sham-operated mice (*n* = 6/group). ^#^*p* < 0.05 *vs.* mice infected with EV-mimic-NC + oe-NC. ^[*dollar*]^*p* < 0.05 *vs.* mice infected with EV-miR-132-mimics + oe-NC. ^&^*p* < 0.05 vs. MCAO mice. An unpaired *t*-test was performed for comparisons of data between two groups. One-way ANOVA was conducted for multiple group comparisons, followed by Tukey’s *post hoc* test.

Following the establishment of the MCAO mouse model, the expression of miR-132 was detected by RT-qPCR. Western blot analysis was subsequently performed to detect protein expression of Acvr2b, Smad2, p-Smad2, and c-jun and results showed that expression of miR-132 in mice infected with EV-mimic-NC + oe-NC was notably downregulated in comparison with that in sham-operated mice, while the expression of Acvr2b and p-Smad2 was significantly increased. Besides, expression of miR-132 in mice infected with EV-miR-132 mimic + oe-NC and infected with EV-miR-132 mimic + oe-c-jun was markedly upregulated in comparison with that in mice infected with EV-mimic-NC + oe-NC, while the levels of Acvr2b and p-Smad2 were markedly decreased. When compared to the sham-operated mice, mice infected with EV-mimic-NC + oe-NC induced highly increased c-jun expression. Compared to mice infected with EV-mimic-NC + oe-NC, c-jun expression in mice infected with EV-miR-132 mimic + oe-NC was downregulated. However, mice infected with EV-miR-132 mimic + oe-c-jun inidcated that the expression of c-jun was significantly increased in the mice infected with EV-miR-132 mimic + oe-NC (Figures 7A,B).

The neurological scores indicated that relative to the sham-operated mice, the mice infected with EV-mimic-NC + oe-NC induced decline in neurological scores. However, neurological scores in mice infected with EV-miR-132 mimic + oe-NC were increased compared to mice infected with EV-mimic-NC + oe-NC. Interestingly, the neurological scores in mice infected with EV-miR-132 mimic + oe-c-jun were decreased compared to mice infected with EV-miR-132 mimic + oe-NC (Figure 7C). The foot fault test results demonstrated that the number of stomping of the right injured limb in mice infected with EV-mimic-NC + oe-NC was notably increased compared with sham-operated mice. However, the number of stomping of right injured limb in mice infected with EV-miR-132 mimic + oe-NC was markedly reduced in comparison with that in mice infected with EV-mimic-NC + oe-NC. In the mice infected with EV-miR-132 mimic + oe-c-jun had a significant increased times compared to that in mice infected with EV-miR-132 mimic + oe-c-jun (Figure 7D). The pole test results revealed that compared with sham-operated mice, time taken for mice to turn 180° head down (T_turn_) and to reach ground (T_total_) was increased in those infected with EV-mimic-NC + oe-NC (*p* < 0.05). In comparison with mice infected with EV-mimic-NC + oe-NC, time taken for mice to turn 180° head down (T_turn_) and to reach ground (T_total_) was markedly declined in mice infected with EV-miR-132 mimic + oe-NC. However, a greater amount of time was required in the mice infected with EV-miR-132 mimic + oe-c-jun compared with mice infected with EV-miR-132 mimic + oe-NC (Figure 7E). The HE staining results revealed that relative to the sham-operated mice, the pathological features in mice infected with EV-mimic-NC + oe-NC was more distinct. In comparison with mice injected with EVsmimic-NC + oe-NC, pathological features in tissues of mice injected with EV-miR-132 mimic + oe-NC were unclear. Compared with mice injected with EV-mir-132 mimic + oe-c-jun, mice injected with EV-mir-132 mimic + oe-NC exhibited notable pathological changes (Figure 7F). The TUNEL staining results revealed that compared to sham-operated mice, mice infected with EV-mimic-NC + oe-NC showed a notable increase in neuron apoptosis. In contrast, neuron apoptosis was strikingly reduced in mice infected with EV-miR-132 mimic + oe-NC, when compared to mice infected with EV-mimic-NC + oe-NC. The rate of apoptosis in the neurons of the hippocampus was increased in mice infected with EV-miR-132 mimic + oe-c-jun when compared to mice infected with EV-miR-132 mimic + oe-NC (Figure 7G). The TTC staining results exhibited that in comparison with sham-operated mice, infarct volume in mice infected with EV-mimic-NC + oe-NC was remarkably augmented. In comparison with mice infected with EV-mimic-NC + oe-NC, infarct volume in mice infected with EV-miR-132 mimic + oe-NC was reduced. The volume of cerebral infarction in mice infected with EV-miR-132 mimic + oe-c-jun was increased when compared to mice infected with EV-miR-132 mimic + oe-NC (Figure 7H). The above-mentioned results demonstrate that EV-derived miR-132 from MSCs could alleviate ischemic neuron injury in MCAO mice by inhibiting the p-Smad2/c-jun signaling pathway.

## Discussion

Cerebral I/R can arise following mechanical thrombectomy as a result of ischemic stroke treatment. Besides, reactive oxygen species produced following reperfusion has been shown to trigger neuronal damage, which consequently leads to disability and death (Sun and Yue, 2019). Cerebral injury triggered by ischemia often leads to neuronal apoptosis. For example, delayed neuronal death in selectively vulnerable regions has been reported, causing irreversible damage (Shao et al., 2017). The relationship between neuronal apoptosis, oxidative stress, and neurodegenerative diseases has been well documented with the enhancement of neuronal survival a crucial target for the treatment of neuronal diseases (Wang M. et al., 2016). Hypoxic stress has been implicated as a cause of neuronal death in various types of brain diseases (Gao et al., 2015). Cell-oriented therapy with MSCs has been highlighted to protect cortical neurons from hypoxic-ischemic damage in stroke patients (Kong et al., 2017). Hence, the current study was designed to elucidate the molecular mechanism of EV-derived miR-132 from MSCs in ischemic neuronal injury through regulation of Acvr2b expression and Smad2/c-jun pathway. A key observation made during our study revealed that EV-derived miR-132 from MSCs exerted a protective effect on ischemic neuronal injury by repressing Acvr2b expression and further inhibiting the p-Smad2/c-jun pathway.

Initially, we detected that Acvr2b was overly expressed in MCAO mice and neurons exposed to OGD conditions. Consistently, Acvr2b has been reported to exhibit high levels of expression in neuronal injury and may promote neuronal injury (Looney et al., 2017). Our results further evidenced that silencing of Acvr2b led to an increase in neuronal activity, decreased neuronal apoptosis, reduced expression of Bax and Cleaved-caspase 3, as well as upregulated Bcl-2 expression, highlighting that inhibition of Acvr2b exerts significant protection against neuronal injury. Consistent with our results, the knockdown of Acvr2b has been reported to contribute to a marked reduction in neuronal apoptosis (Koszinowski et al., 2015; Magga et al., 2019). Moreover, we expounded that miR-132 targeted and inhibited expression of Acvr2b. Acvr2b has been previously predicted to be one of the downstream targets of miR-132 (Jiang et al., 2015). Downregulation of miR-132 expression has been identified in MCAO mice and OGD-treated neurons, while upregulation of miR-132 has been speculated to diminish brain damage in MCAO mice and protect hippocampal neurons against OGD-induced apoptosis (Mazziotti et al., 2017; Zuo et al., 2019). Moreover, the overexpression of miR-132 has been shown to contribute to enhanced neuronal viability (Pan et al., 2020), decreased neuronal apoptosis, and elevated expression of Bcl-2 while downregulated levels of Bax as well as Caspase-3 have been reported following OGD treatment (Sun et al., 2017). As described reported, BMSCs-released EVs can improve ischemic neuronal injury and EVs are abundant in miR-132 (Xiao et al., 2018). Hence, our results indicated that EV-derived miR-132 from MSCs could attenuate neuronal damage by repressing expression of Acvr2b.

In subsequent experiments, our study revealed that Acvr2b promotes activation of a p-Smad2/c-jun signaling pathway to induce neuronal injury. A previous study concluded that Acvr2b promotes p-Smad2 level (Gao et al., 2018) and p-Smad2 elevates expression of c-jun (Thakur et al., 2014). Significant upregulation of p-Smad2 and c-jun expression has been emphasized in cerebral I/R injury and higher expression of p-Smad2 and c-jun induce neuronal injury (Wang S. et al., 2016). Furthermore, our findings provided evidence suggesting that the overexpression of c-jun abrogated protective role of EV-derived miR-132 from MSCs in neurons as increased c-jun expression induced aggravated neuronal apoptosis and neuronal injury. Existing literature, the overexpression of c-jun contributes to neuronal apoptosis which is evidently observed in neurodegenerative diseases such as Alzheimer’s and dementia, as well as brain damage, including stroke and epilepsy (Kravchick et al., 2016). In neuronal cells, activation of c-JNK pathway has also been demonstrated to induce cell damage (Minamiyama et al., 2012).

## Conclusion

**FIGURE 8:**
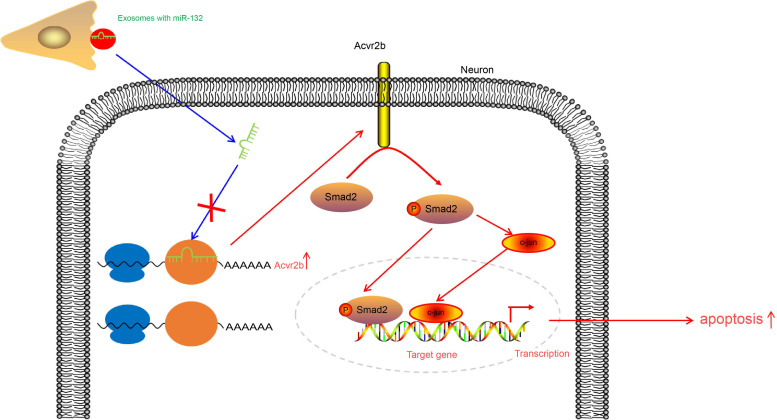
EV-derived miR-132 released from MSCs improves ischemic neuronal injury by targeting and repressing Acvr2b *via* inhibiting p-Smad2/c-jun pathway.

Taken together, the key findings of the current study present evidence demonstrating that upregulation of miR-132-containing EVs released from MSCs was capable of ameliorating ischemic neuronal injury through suppression of Acvr2b expression *via* inhibition of p-Smad2/c-jun pathway (Figure 8). Our findings provide a theoretical basis as well as a fresh perspective onto novel therapeutic strategies of ischemic neuronal injury treatment. Prospective studies for translation from mechanism and animal experiments to clinical applications are required in order to further characerize the role of EV-derived miR-132 from MSCs in this disorder. Future investigations will be aimed at identifying the optimum dose and time for EVs injection.

## Data Availability Statement

The original contributions presented in the study are included in the article/Supplementary Material, further inquiries can be directed to the corresponding author/s.

## Ethics Statement

The studies involving human participants were reviewed and approved by Shandong Provincial Hospital. Written informed consent for participation was not required for this study in accordance with the national legislation and the institutional requirements. The animal study was reviewed and approved by Shandong Provincial Hospital.

## Author Contributions

BF, LM, LL, and ZF participated in the conception and design of the study. BF, LM, ZF, and PZ performed the analysis and interpretation of data. BF, LL, and ZF contributed to drafting the article. BF and PZ revised it critically for important intellectual content. GZ is the GUARANTOR for the article who accepts full responsibility for the work and/or the conduct of the study, had access to the data, and oversaw the decision to publish. All authors contributed to the article and approved the submitted version.

## Conflict of Interest

The authors declare that the research was conducted in the absence of any commercial or financial relationships that could be construed as a potential conflict of interest.

## Supplementary Material

Supplementary Figure 1 sh-Acvr2b-1 is selected for subsequent experiments. **(A)** Silencing efficiency of Acvr2b mRNA in OGD-exposed neurons examined using RT-qPCR. **(B)** The protein expression of Acvr2b silencing efficiency in OGD-exposed neurons examined using western blot analysis normalized to GAPDH. #*p* < 0.05 *vs.* sh-NC transfected neurons. One-way ANOVA was conducted for multiple group comparisons, followed by Tukey’s *post hoc* test.

Supplementary Figure 2 The transfection efficiency of miR-132 mimic in HEK293T cells and neurons assessed by RT-qPCR. **(A)** The transfection efficiency of miR-132 mimic in HEK293T cells. **(B)** The transfection efficiency of miR-132 mimic in neurons. ^∗^*p* < 0.05 *vs.* mimic-NC-transfected HEK293T cells or neurons. An unpaired *t*-test was performed for comparisons of data between two groups.

Supplementary Figure 3 The transfection efficiency of miR-132 mimic in BMSCs and BMSC-derived EVs. **(A)** The expression of miR-132 in BMSCs after overexpression of miR-132 detected by RT-qPCR. **(B)** The expression of miR-132 in EVs after overexpression of miR-132 from BMSCs detected by RT-qPCR. ^∗^*p* < 0.05 *vs.* mimic-NC transfected BMSCs, EV-mimic-NC treated neurons or normal controls. An unpaired *t*-test was performed for comparisons of data between two groups.

## Abbreviations

Acvr2b: activin receptor type IIB
ANOVA: analysis of variance
CANX: calnexin
CCK-8: Cell Counting kit-8
DLS: dynamic light scattering
DMEM: Dulbecco’s modified eagle medium
EV: extracellular vesicles
FBS: fetal bovine serum
FITC: fluorescein isothiocyanate
GAPDH: glyceraldehyde-3-phosphate dehydrogenase
HBSS: Hank’s balanced salt solution
HE: hematoxylin and eosin
HIE: hypoxic-ischemic encephalopathy
I/R: ischemia and reperfusion
JNK: Jun n-terminal kinase
LDH: lactate dehydrogenase
MCAO: middle cerebral artery occlusion
miRs: microRNAs
MSC: mesenchymal stem cell
oe: overexpressed
OGD: oxygen and glucose deprivation
PDL: periodontal ligament
PI: propidium iodide
p-Smad 2: phosphorylated-Smad2
TBST: tris-buffered saline Tween
TEM: transmission electron microscope
TGF- β: transforming growth factor- β
TTC: 2,3,5-triphenyltetrazolium hydrochloride
TUNEL: terminal deoxynucleotidyl transferase-mediated dUTP nick end-labeling
UTR: untranslated region.

## References

[B1] Chakraborty A. Diefenbacher M. E. Mylona A. Kassel O. Behrens A. (2015). The E3 ubiquitin ligase Trim7 mediates c-Jun/AP-1 activation by Ras signalling. *Nat. Commun.* 6:6782. 10.1038/ncomms7782 25851810 PMC4395875

[B2] Du W. Huang J. Yao H. Zhou K. Duan B. Wang Y. (2010). Inhibition of TRPC6 degradation suppresses ischemic brain damage in rats. *J. Clin. Invest.* 120 3480–3492. 10.1172/JCI43165 20811149 PMC2947234

[B3] Feng D. Wang B. Wang L. Abraham N. Tao K. Huang L. (2017). Pre-ischemia melatonin treatment alleviated acute neuronal injury after ischemic stroke by inhibiting endoplasmic reticulum stress-dependent autophagy via PERK and IRE1 signalings. *J. Pineal. Res.* 62:12395. 10.1111/jpi.12395 28178380

[B4] Gao C. Zhu W. Tian L. Zhang J. Li Z. (2015). MCT4-mediated expression of EAAT1 is involved in the resistance to hypoxia injury in astrocyte-neuron co-cultures. *Neurochem. Res.* 40 818–828. 10.1007/s11064-015-1532-2 25645447

[B5] Gao X. Zhao P. Hu J. Zhu H. Zhang J. Zhou Z. (2018). MicroRNA-194 protects against chronic hepatitis B-related liver damage by promoting hepatocyte growth via ACVR2B. *J. Cell. Mol. Med.* 22 4534–4544. 10.1111/jcmm.13714 30044042 PMC6111826

[B6] Garcia J. H. Wagner S. Liu K. F. Hu X. J. (1995). Neurological deficit and extent of neuronal necrosis attributable to middle cerebral artery occlusion in rats. Statistical validation. *Stroke* 26 627–634; discussion 635. 10.1161/01.str.26.4.627 7709410

[B7] Gotoh T. Iwahana H. Kannan S. Marei R. G. Mousa H. Elgamal M. (2020). Glycosylation is a novel TGFbeta1-independent post-translational modification of Smad2. *Biochem. Biophys. Res. Commun.* 521 1010–1016. 10.1016/j.bbrc.2019.11.039 31727370

[B8] Jiang X. Chen X. Chen L. Ma Y. Zhou L. Qi Q. (2015). Upregulation of the miR-212/132 cluster suppresses proliferation of human lung cancer cells. *Oncol. Rep.* 33 705–712. 10.3892/or.2014.3637 25435090

[B9] Kong D. Zhu J. Liu Q. Jiang Y. Xu L. Luo N. (2017). Mesenchymal stem cells protect neurons against hypoxic-ischemic injury via inhibiting parthanatos, necroptosis, and apoptosis, but not autophagy. *Cell. Mol. Neurobiol.* 37 303–313. 10.1007/s10571-016-0370-3 27044018 PMC11482119

[B10] Koritzinsky E. H. Street J. M. Star R. A. Yuen P. S. (2017). Quantification of exosomes. *J. Cell Physiol.* 232 1587–1590. 10.1002/jcp.25387 27018079 PMC5039048

[B11] Koszinowski S. Buss K. Kaehlcke K. Krieglstein K. (2015). Signaling via the transcriptionally regulated activin receptor 2B is a novel mediator of neuronal cell death during chicken ciliary ganglion development. *Int. J. Dev. Neurosci.* 41 98–104. 10.1016/j.ijdevneu.2015.01.006 25660516

[B12] Kou X. Xu X. Chen C. Sanmillan M. L. Cai T. Zhou Y. (2018). The Fas/Fap-1/Cav-1 complex regulates IL-1RA secretion in mesenchymal stem cells to accelerate wound healing. *Sci. Transl. Med.* 10:eaai8524. 10.1126/scitranslmed.aai8524 29540618 PMC6310133

[B13] Kravchick D. O. Karpova A. Hrdinka M. Lopez-Rojas J. Iacobas S. Carbonell A. U. (2016). Synaptonuclear messenger PRR7 inhibits c-Jun ubiquitination and regulates NMDA-mediated excitotoxicity. *EMBO J.* 35 1923–1934. 10.15252/embj.201593070 27458189 PMC5007554

[B14] Launey Y. Fryer T. D. Hong Y. T. Steiner L. A. Nortje J. Veenith T. V. (2020). Spatial and temporal pattern of ischemia and abnormal vascular function following traumatic brain injury. *JAMA Neurol.* 77 339–349. 10.1001/jamaneurol.2019.3854 31710336 PMC6865302

[B15] Lin S. I. Lin L. D. Chang H. H. Chang M. C. Wang Y. L. Pan Y. H. (2018). IL-1beta induced IL-8 and uPA expression/production of dental pulp cells: role of TAK1 and MEK/ERK signaling. *J. Formos Med. Assoc.* 117 697–704. 10.1016/j.jfma.2018.04.003 29709340

[B16] Looney A. M. Ahearne C. E. Hallberg B. Boylan G. B. Murray D. M. (2017). Downstream mRNA target analysis in neonatal hypoxic-ischaemic encephalopathy identifies novel marker of severe injury: a proof of concept paper. *Mol. Neurobiol.* 54 8420–8428. 10.1007/s12035-016-0330-4 27957679

[B17] Luo P. Fu X. Chang M. Zhang L. Guo L. (2020). Cerebral ischemia-reperfusion causes a down regulation of HCN1 expression via enhancing the nuclear NRSF-HDAC4 gathering that contributes to neuron damage. *Brain Res. Bull.* 156 50–57. 10.1016/j.brainresbull.2020.01.001 31923455

[B18] Ma T. Chen Y. Chen Y. Meng Q. Sun J. Shao L. (2018). MicroRNA-132, delivered by mesenchymal stem cell-derived exosomes, promote angiogenesis in myocardial infarction. *Stem Cells Int.* 2018:3290372. 10.1155/2018/3290372 30271437 PMC6151206

[B19] Magga J. Vainio L. Kilpio T. Hulmi J. J. Taponen S. Lin R. (2019). Systemic blockade of ACVR2B ligands protects myocardium from acute ischemia-reperfusion injury. *Mol. Ther.* 27 600–610. 10.1016/j.ymthe.2019.01.013 30765322 PMC6404100

[B20] Mai C. Mankoo H. Wei L. An X. Li C. Li D. (2020). TRPM2 channel: a novel target for alleviating ischaemia-reperfusion, chronic cerebral hypo-perfusion and neonatal hypoxic-ischaemic brain damage. *J. Cell. Mol. Med.* 24 4–12. 10.1111/jcmm.14679 31568632 PMC6933339

[B21] Mazziotti R. Baroncelli L. Ceglia N. Chelini G. Sala G. D. Magnan C. (2017). Mir-132/212 is required for maturation of binocular matching of orientation preference and depth perception. *Nat. Commun.* 8:15488. 10.1038/ncomms15488 28534484 PMC5457514

[B22] Minamiyama M. Katsuno M. Adachi H. Doi H. Kondo N. Iida M. (2012). Naratriptan mitigates CGRP1-associated motor neuron degeneration caused by an expanded polyglutamine repeat tract. *Nat. Med.* 18 1531–1538. 10.1038/nm.2932 23023499

[B23] Molcho L. Ben-Zur T. Barhum Y. Angel A. Glat M. Offen D. (2018). Combined gene therapy to reduce the neuronal damage in the mouse model of focal ischemic injury. *J. Mol. Neurosci.* 66 180–187. 10.1007/s12031-018-1143-x 30178388

[B24] Nyati S. Schinske K. Ray D. Nyati M. K. Ross B. D. Rehemtulla A. (2011). Molecular imaging of TGFbeta-induced Smad2/3 phosphorylation reveals a role for receptor tyrosine kinases in modulating TGFbeta signaling. *Clin. Cancer Res.* 17 7424–7439. 10.1158/1078-0432.CCR-11-1248 21948232 PMC3229686

[B25] Olsen O. E. Wader K. F. Hella H. Mylin A. K. Turesson I. Nesthus I. (2015). Activin A inhibits BMP-signaling by binding ACVR2A and ACVR2B. *Cell Commun. Signal.* 13 27. 10.1186/s12964-015-0104-z 26047946 PMC4467681

[B26] Pan Q. Kuang X. Cai S. Wang X. Du D. Wang J. (2020). miR-132-3p priming enhances the effects of mesenchymal stromal cell-derived exosomes on ameliorating brain ischemic injury. *Stem Cell Res. Ther.* 11:260. 10.1186/s13287-020-01761-0 32600449 PMC7322840

[B27] Saugstad J. A. (2010). MicroRNAs as effectors of brain function with roles in ischemia and injury, neuroprotection, and neurodegeneration. *J. Cereb. Blood Flow Metab.* 30 1564–1576. 10.1038/jcbfm.2010.101 20606686 PMC2932764

[B28] Sessa F. Maglietta F. Bertozzi G. Salerno M. Di Mizio G. Messina G. (2019). Human brain injury and miRNAs: an experimental study. *Int. J. Mol. Sci.* 20:1546. 10.3390/ijms20071546 30934805 PMC6479766

[B29] Shao S. Xu M. Zhou J. Ge X. Chen G. Guo L. (2017). Atorvastatin attenuates ischemia/reperfusion-induced hippocampal neurons injury Via Akt-nNOS-JNK signaling pathway. *Cell. Mol. Neurobiol.* 37 753–762. 10.1007/s10571-016-0412-x 27488855 PMC11482104

[B30] Sun J. Yue F. (2019). Suppression of REDD1 attenuates oxygen glucose deprivation/reoxygenation-evoked ischemic injury in neuron by suppressing mTOR-mediated excessive autophagy. *J. Cell. Biochem.* 120 14771–14779. 10.1002/jcb.28737 31021470

[B31] Sun Z. Z. Lv Z. Y. Tian W. J. Yang Y. (2017). MicroRNA-132 protects hippocampal neurons against oxygen-glucose deprivation-induced apoptosis. *Int. J. Immunopathol. Pharmacol.* 30 253–263. 10.1177/0394632017715837 28627974 PMC5815264

[B32] Thakur N. Gudey S. K. Marcusson A. Fu J. Y. Bergh A. Heldin C. H. (2014). TGFbeta-induced invasion of prostate cancer cells is promoted by c-Jun-dependent transcriptional activation of Snail1. *Cell Cycle* 13 2400–2414. 10.4161/cc.29339 25483191 PMC4128885

[B33] Wang M. Li Y. J. Ding Y. Zhang H. N. Sun T. Zhang K. (2016). Silibinin prevents autophagic cell death upon oxidative stress in cortical neurons and cerebral ischemia-reperfusion injury. *Mol. Neurobiol.* 53 932–943. 10.1007/s12035-014-9062-5 25561437

[B34] Wang S. Yin J. Ge M. Dai Z. Li Y. Si J. (2016). Transforming growth-beta 1 contributes to isoflurane postconditioning against cerebral ischemia-reperfusion injury by regulating the c-Jun N-terminal kinase signaling pathway. *Biomed Pharmacother.* 78 280–290. 10.1016/j.biopha.2016.01.030 26898453

[B35] Xiao Y. Geng F. Wang G. Li X. Zhu J. Zhu W. (2018). Bone marrow-derived mesenchymal stem cells-derived exosomes prevent oligodendrocyte apoptosis through exosomal miR-134 by targeting caspase-8. *J. Cell. Biochem.* 120:27519. 10.1002/jcb.27519 30191592

[B36] Xu B. Qin Y. Li D. Cai N. Wu J. Jiang L. (2020). Inhibition of PDE4 protects neurons against oxygen-glucose deprivation-induced endoplasmic reticulum stress through activation of the Nrf-2/HO-1 pathway. *Redox Biol.* 28:101342. 10.1016/j.redox.2019.101342 31639651 PMC6807264

[B37] Yu H. Kalogeris T. Korthuis R. J. (2019). Reactive species-induced microvascular dysfunction in ischemia/reperfusion. *Free Radic. Biol. Med.* 135 182–197. 10.1016/j.freeradbiomed.2019.02.031 30849489 PMC6503659

[B38] Zhang H. Wu J. Wu J. Fan Q. Zhou J. Wu J. (2019). Exosome-mediated targeted delivery of miR-210 for angiogenic therapy after cerebral ischemia in mice. *J. Nanobiotechnol.* 17:29. 10.1186/s12951-019-0461-7 30782171 PMC6379944

[B39] Zhang N. Zhong J. Han S. Li Y. Yin Y. Li J. (2016). MicroRNA-378 alleviates cerebral ischemic injury by negatively regulating apoptosis executioner caspase-3. *Int. J. Mol. Sci.* 17:1427. 10.3390/ijms17091427 27598143 PMC5037706

[B40] Zuo X. Lu J. Manaenko A. Qi X. Tang J. Mei Q. (2019). MicroRNA-132 attenuates cerebral injury by protecting blood-brain-barrier in MCAO mice. *Exp. Neurol.* 316 12–19. 10.1016/j.expneurol.2019.03.017 30930097

