# Supplementary figures and images for: Upregulation of Extracellular Vesicles-Encapsulated miR-132 Released From Mesenchymal Stem Cells Attenuates Ischemic Neuronal Injury by Inhibiting Smad2/c-jun Pathway via Acvr2b Suppression
Source: Front Cell Dev Biol. 2021 Mar 8;8:568304. doi: 10.3389/fcell.2020.568304 (PMC7982537; doi:10.3389/fcell.2020.568304)

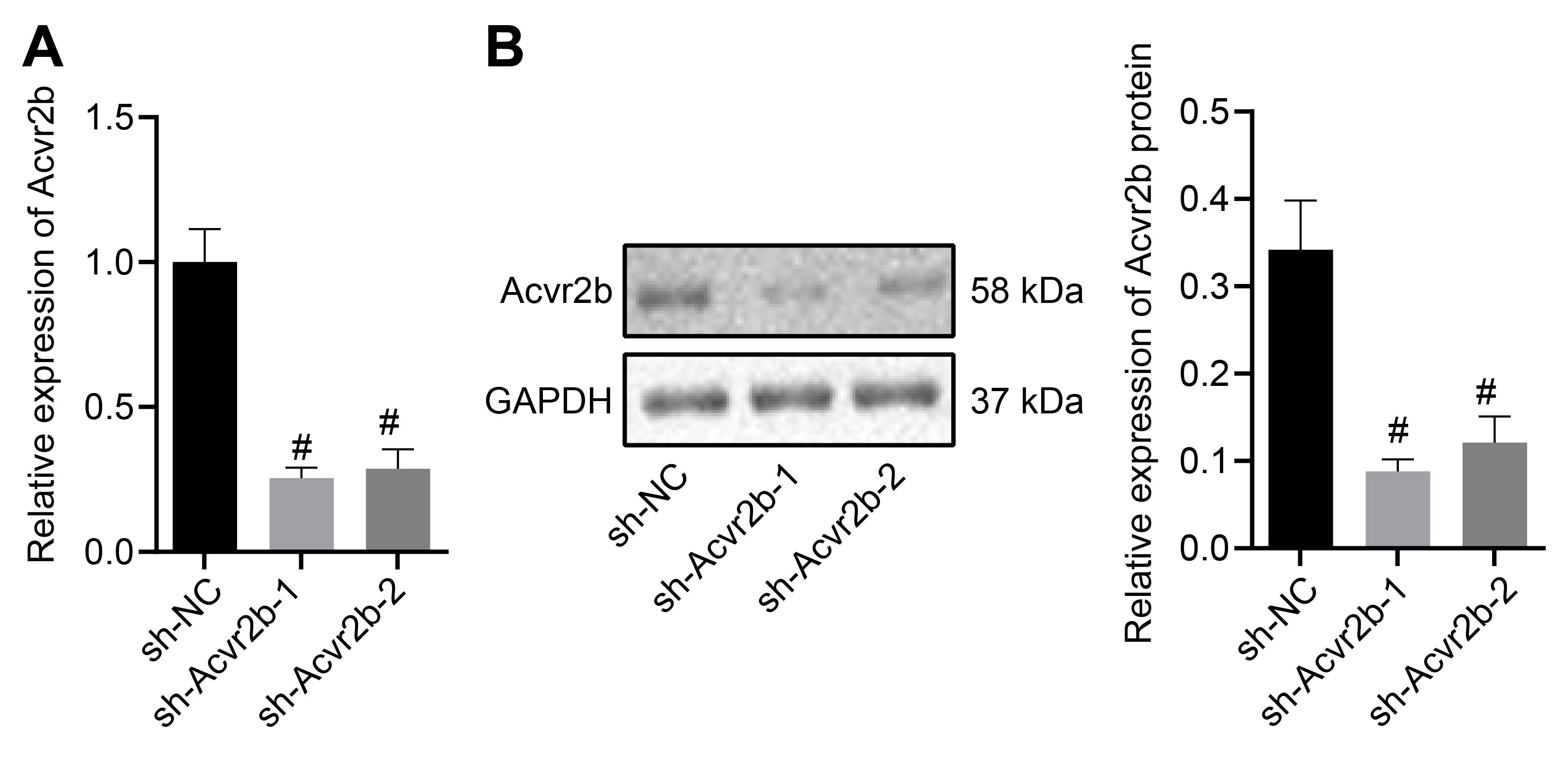

Supplement: Supplementary Figure 1 — sh-Acvr2b-1 is selected for subsequent experiments. (A) Silencing efficiency of Acvr2b mRNA in OGD-exposed neurons examined using RT-qPCR. (B) The protein expression of Acvr2b silencing efficiency in OGD-exposed neurons examined using western blot analysis normalized to GAPDH. #p < 0.05 vs. sh-NC transfected neurons. One-way ANOVA was conducted for multiple group comparisons, followed by Tukey’s post hoc test. [file Image_1.JPEG]

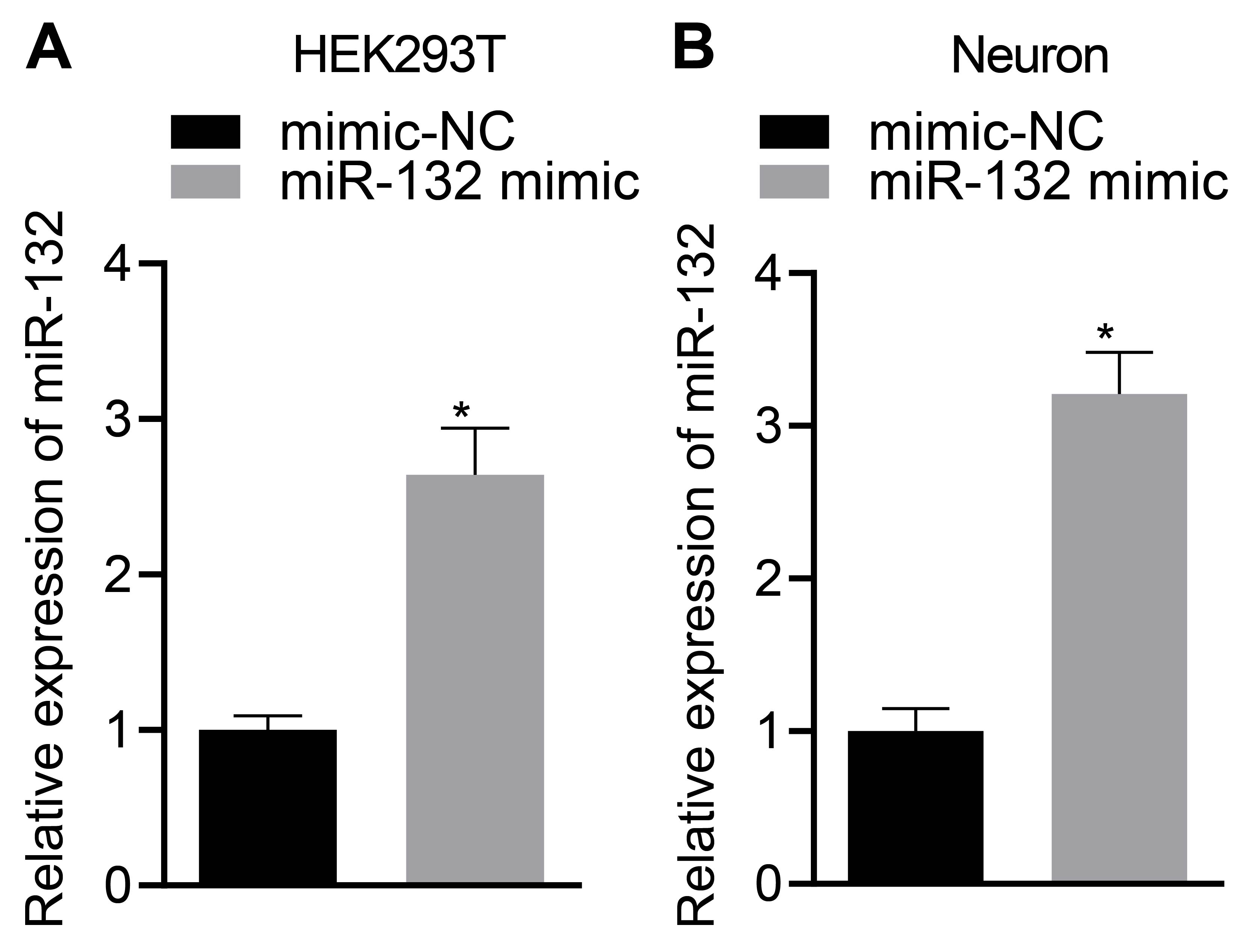

Supplement: Supplementary Figure 2 — The transfection efficiency of miR-132 mimic in HEK293T cells and neurons assessed by RT-qPCR. (A) The transfection efficiency of miR-132 mimic in HEK293T cells. (B) The transfection efficiency of miR-132 mimic in neurons. ∗p < 0.05 vs. mimic-NC-transfected HEK293T cells or neurons. An unpaired t-test was performed for comparisons of data between two groups. [file Image_2.JPEG]

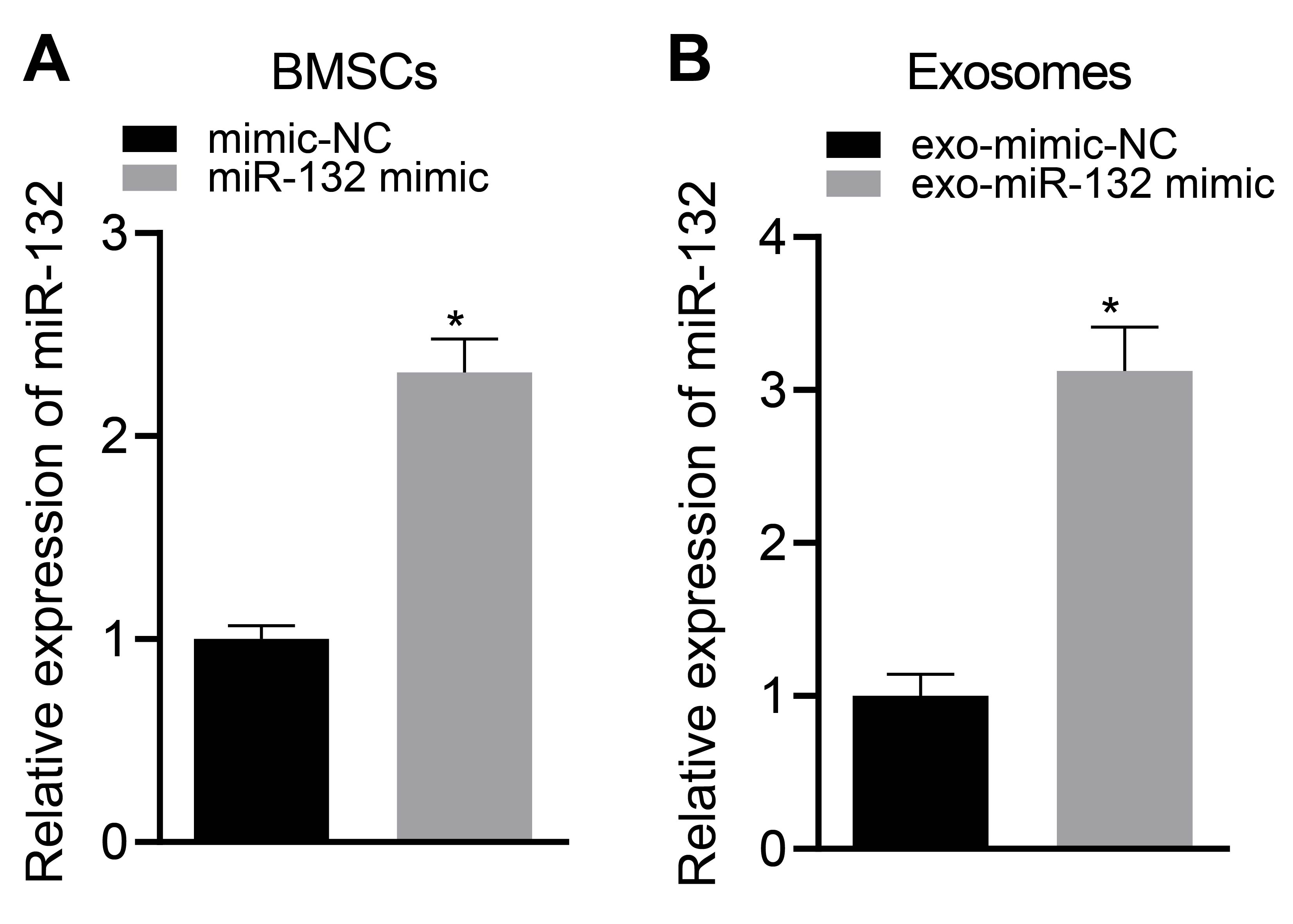

Supplement: Supplementary Figure 3 — The transfection efficiency of miR-132 mimic in BMSCs and BMSC-derived EVs. (A) The expression of miR-132 in BMSCs after overexpression of miR-132 detected by RT-qPCR. (B) The expression of miR-132 in EVs after overexpression of miR-132 from BMSCs detected by RT-qPCR. ∗p < 0.05 vs. mimic-NC transfected BMSCs, EV-mimic-NC treated neurons or normal controls. An unpaired t-test was performed for comparisons of data between two groups. [file Image_3.JPEG]
